# An Adaptive Multi-Representation Fusion Framework for EMG Pattern Recognition in Transradial Amputees

**DOI:** 10.3390/bioengineering13080900

**Published:** 2026-08-09

**Authors:** Ejay Nsugbe, Oluwarotimi W. Samuel

**Affiliations:** 1 Nsugbe Research Labs, Swindon SN1 3LG, UK; 2 Data Science Research Centre and School of Computing, College of Science and Engineering, University of Derby, Derby DE22 3AW, UK

**Keywords:** adaptive signal decomposition, electromyography (EMG), information fusion, myoelectric pattern recognition, prosthetic control, surface electromyography

## Abstract

Electromyography (EMG)-based pattern recognition is a key enabling technology for intuitive upper-limb prosthetic control; however, amputee EMG signals show substantial intersubject variability and nonstationary characteristics, which can limit the effectiveness of conventional fixed feature representations. This study proposes an Adaptive Multi-Representation Fusion Framework that combines an enhanced Adaptive Linear Series Decomposition Learner (Adaptive LSDL) with Time-Domain Power Spectral Descriptors (TDPSD) for amputee movement classification. The proposed Adaptive LSDL extends conventional decomposition by using adaptive threshold-region optimization and multitransform signal analysis, enabling subject-specific identification of the most discriminative decomposition characteristics while preserving low computational complexity. The complementary properties of TDPSD and Adaptive LSDL are then exploited through feature-level fusion, producing a unified representation that captures both local temporal-spectral complexity and adaptive structural signal information. Experimental evaluation was conducted using the Ninapro DB3 dataset, comprising 11 transradial amputees, and a MYO armband amputee dataset, comprising 6 transradial amputees. Subject-specific five-fold cross-validation was performed using a Linear Discriminant Analysis (LDA) classifier. Adaptive LSDL consistently improved performance relative to the original fixed-threshold LSDL representation, while fusion improved classification accuracy for 7 of 11 DB3 amputees and 5 of 6 MYO amputees. The largest fusion gains reached 4.39 and 5.60 percentage points for the DB3 and MYO cohorts, respectively. Furthermore, the proposed framework demonstrated competitive performance relative to a lightweight real-time convolutional neural network benchmark while requiring substantially lower computational complexity. These findings indicate that adaptive decomposition and representation-level information fusion provide an effective approach for exploiting subject-specific neuromuscular information while remaining suitable for computationally efficient prosthetic control applications.

## 1. Introduction

### 1.1. Background and Motivation

Surface electromyography (sEMG) has become one of the most widely studied bio-signals for intuitive myoelectric control of upper-limb prostheses [1]. Its operating principle is based on the measurement of electrical activity generated during muscle contraction [2]. Accordingly, sEMG provides a non-invasive interface through which movement intentions can be decoded and translated into prosthetic gesture commands [1]. Further advances in machine learning and pattern recognition have substantially improved the reliability of sEMG-based control systems, enabling the recognition of increasingly complex movement classes and facilitating more natural human–machine interaction [1,3].

Despite these advances, several challenges remain in this research area, as amputees typically exhibit sEMG signals with reduced signal quality, altered muscle activation patterns, physiological variability, electrode displacement, and substantial inter-subject heterogeneity [1]. From a signal perspective, these factors introduce non-stationarity into the recorded signals and can degrade classification performance when conventional feature extraction methods are used [4]. This situation warrants the development of innovative signal processing methods and feature extraction methods, which remain central challenges in myoelectric pattern recognition [1].

Related studies have shown that pattern recognition systems often rely on a single feature representation extracted from the signal domain, where time-domain descriptors, frequency-domain features, wavelet coefficients, and nonlinear measures have all demonstrated effectiveness under specific conditions [5]. However, due to the complexity and variability of amputee neuromuscular architectures, it is recognized that no single representation can fully characterize the neuromuscular information embedded within EMG signals [1]. Thus, reliance on a single representation may limit the amount of discriminative information available to the classification model, particularly in challenging amputee datasets where signal variability is pronounced [1,5]. These limitations have motivated the exploration of multi-representation learning strategies capable of integrating complementary information extracted from different feature spaces [6,7]. This fusion process involves combining heterogeneous representations of the same physiological signal, which may provide a more complete characterization of the underlying neuromuscular motor output and may improve robustness and classification accuracy while maintaining computational efficiency suitable for real-time prosthetic deployment [6,8]. Collectively, these challenges suggest that improving EMG pattern recognition in amputees may depend not only on developing stronger individual feature representations, but also on determining whether different representations capture complementary aspects of the underlying neuromuscular signal. This study therefore examines whether integrating a well-established temporal descriptor with an adaptive decomposition-based representation can utilize complementary information to improve movement classification while preserving the computational efficiency required for real-time prosthetic control.

**Figure 1:**
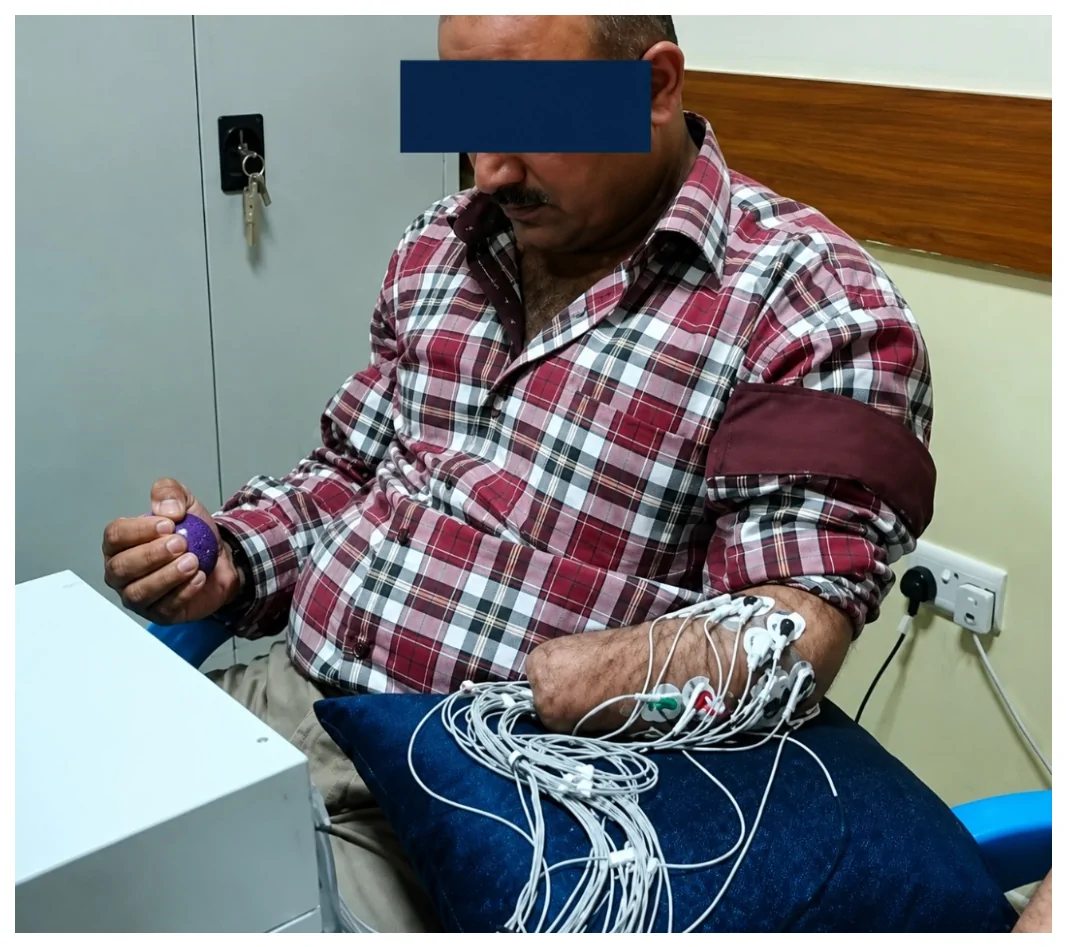
An image of an amputee during a signal acquisition process [9].

Figure 1 shows a sample transradial amputee with EMG sensors during a gesture recognition exercise.

### 1.2. Information Fusion Perspective

Information fusion seeks to improve decision-making by integrating complementary information from multiple sources, modalities, or representations [10,11]. Although fusion is often performed across different sensors, an equally important paradigm is the fusion of heterogeneous representations derived from the same sensing modality [6,11]. In this context, distinct feature extraction techniques may be treated as alternative observations of the underlying physiological process, each encoding unique discriminative characteristics [1,5].

In the EMG signal processing literature, the Time-Domain Power Spectral Descriptors (TDPSD) proposed by Khushaba et al. have emerged as an effective representation of EMG signals because they capture signal energy distribution and nonlinear amplitude characteristics while maintaining low computational complexity [12,13]. The TDPSD features have demonstrated strong performance across a range of myoelectric classification tasks and remain suitable for real-time embedded implementations [9,12,13]. However, like all fixed feature representations, TDPSD emphasizes specific signal characteristics and may not fully capture the diverse temporal structures present in amputee EMG recordings [1,6].

At the same time, signal decomposition methods have shown promise in signal processing problems because, prior to feature extraction, decomposition can reduce signal redundancy and preserve regions with maximal information content, much like the Wavelet Transform [14,15]. The Linear Series Decomposition Learner (LSDL) is a multiresolution signal decomposition approach whose conceptual origins derive from seminal contributions by Nsugbe et al. in acoustic emission analysis for estimating particle sizes in heterogeneous powder mixtures [16,17]. Working in the time domain, its architecture uses a series of tuned linear basis functions and metaheuristic optimization to iteratively decompose a candidate signal into sub-decomposition bands and identify an optimal signal region prior to feature extraction. The approach has also been benchmarked against the wavelet transform in numerous studies, and LSDL decomposition has been shown to exceed the predictive performance of the wavelet transform [6]. Furthermore, the application of LSDL in physiological signal processing has enabled novel signal modelling capabilities in areas such as upper-limb prostheses, cancer, psychiatry, and pregnancy, among others [6].

The main limitation of the method lies in the static assignment of an optimal threshold region. Once a region with maximal information content is identified, the corresponding signal segment is used for feature extraction unless the procedure is repeated from the beginning as part of a region re-selection process. This limitation makes the approach relatively static for dynamic applications, in which the signal source may vary and render the preselected optimal region suboptimal in practice. To address this issue, an adaptive framework has been incorporated into the signal processing architecture of LSDL, allowing it to relearn the optimal region of the signal before making predictive inferences and thereby adapt to the dynamic nature of the signal and its source composition. The original method relied solely on information from the raw, time-domain, amplitude-based signal. As part of the present enhancement, additional computationally efficient signal transformation domains have been integrated into the pipeline, including the signal gradient transformation, curvature transformation, and energy-based transformation [18,19,20]. Together with the amplitude representation, these can be fused to provide a more informative basis for extracting additional signal information while preserving the computational efficiency central to the original LSDL framework.

Thus, the central hypothesis of this work is that TDPSD and Adaptive LSDL provide complementary representations of EMG activity. TDPSD characterizes signal behavior through established power-spectral and amplitude-related descriptors, whereas Adaptive LSDL emphasizes discriminative signal-structure features through multiple representations and adaptive learning. Because these representations encode different aspects of the same neuromuscular phenomenon, their integration constitutes a representation-level information fusion problem. By combining both feature spaces within a unified framework, it becomes possible to exploit complementary information that may not be accessible through either representation alone.

### 1.3. Contributions

The primary contributions of this work are as follows:

1. We introduce Adaptive LSDL, an enhanced adaptive signal representation framework that extends the conventional LSDL methodology through dynamic localization, region optimization, and multi-transform signal analysis, thereby enabling subject-specific extraction of discriminative information from amputee sEMG signals.
2. We investigate the hypothesis that Adaptive LSDL and Time-Domain Power Spectral Descriptors (TDPSD) encode complementary neuromuscular information. To test this hypothesis, we propose a representation-level information fusion framework that integrates both feature spaces and evaluates whether their combination provides improved movement recognition beyond either representation alone. This framework is comprehensively validated across all 11 transradial amputees in the Ninapro DB3 dataset and 6 transradial amputees in the MYO EMG dataset, providing a rigorous assessment under clinically relevant conditions.
3. We benchmark the proposed framework against conventional handcrafted representations and the Real-Time Temporal Convolutional Neural Network (RT-TCNN), demonstrating competitive classification performance while maintaining substantially lower computational complexity.
4. We demonstrate the feasibility of low-complexity, real-time deployment through the use of a lightweight Linear Discriminant Analysis (LDA) classifier, supporting the translation of the proposed framework toward embedded myoelectric prosthetic control systems.

## 2. Related Work

### 2.1. Single-Representation EMG Pattern Recognition

Early myoelectric pattern-recognition systems were predominantly based on handcrafted feature representations extracted from sEMG signals [4,21]. Time-domain descriptors, such as Mean Absolute Value (MAV), Waveform Length (WL), Zero Crossings (ZC), and Slope Sign Changes (SSC), were widely adopted because of their computational simplicity and suitability for real-time prosthetic control [5,21]. Collectively termed the Hudgins feature set, these descriptors showed that meaningful movement-related information could be extracted directly from EMG morphology while preserving low computational cost [4,21].

To improve class discrimination, researchers subsequently introduced frequency-domain and time-frequency representations, including Fourier-based descriptors and wavelet transform coefficients. These approaches enabled the characterization of the nonstationary behaviour inherent in EMG signals and showed improved robustness for complex gesture-recognition tasks. However, their performance is often affected by decomposition parameters, basis selection, and higher computational complexity.

More recently, advanced handcrafted representations, such as entropy-based measures, fractal descriptors, and TDPSD, have been proposed [5]. Among these methods, TDPSD has emerged as one of the best-performing EMG feature families because it can capture signal energy distribution and nonlinear amplitude characteristics while maintaining computational efficiency [9,12,13]. Consequently, TDPSD remains a common benchmark representation for modern myoelectric pattern-recognition systems [5,9].

Despite their differences, these methods share a common assumption: the discriminative information required for classification can be adequately represented by a single signal representation. Although effective under controlled conditions, this assumption may be limiting for amputee datasets, where substantial physiological variability and altered neuromuscular activation patterns are present.

The emergence of deep learning has shifted EMG pattern recognition toward data-driven representation learning [3,7]. Convolutional neural networks (CNNs), recurrent neural networks (RNNs), long short-term memory (LSTM) architectures, and temporal convolutional networks (TCNs) have all shown the ability to learn discriminative representations directly from raw EMG recordings [7,22,23].

CNN-based architectures have been particularly effective because they capture local temporal structure and inter-channel relationships [3,7]. More recently, lightweight temporal architectures have been proposed specifically for prosthetic control applications, where inference latency and computational constraints remain important deployment considerations [8,23]. These approaches aim to balance classification accuracy with practical requirements, including memory footprint, processing speed, and feasibility for embedded implementation [7,8].

Although deep learning approaches have achieved competitive performance across numerous EMG datasets, several challenges remain [3,7]. High-capacity models often require substantial training data, extensive hyperparameter optimisation, and greater computational resources [24]. These limitations are particularly relevant in amputee populations, where data availability is often limited and subject-specific variability remains pronounced [1,4]. Furthermore, the performance gains obtained with increasingly complex architectures do not necessarily indicate that the underlying signal representation has been adequately characterised [5,7].

Consequently, there is still substantial interest in developing compact yet informative signal representations that can achieve competitive performance while preserving the computational efficiency required for real-time prosthetic deployment.

### 2.2. Representation-Level Information Fusion in Biosignal Analysis

Information fusion has traditionally focused on combining complementary information from multiple sensors or modalities [10,11]. In bio-signal processing, this approach has commonly taken the form of multimodal fusion strategies that combine EMG, EEG, inertial measurement units (IMUs), mechanomyography (MMG), near-infrared spectroscopy (NIRS), and other physiological sensing modalities [25,26].

Although multimodal fusion has shown performance gains, an equally important yet comparatively underexamined paradigm is representation-level fusion [11,27]. In this framework, multiple feature representations extracted from the same sensing modality are treated as complementary observations of the underlying physiological process [10,27]. Rather than relying on a single representation, the goal is to integrate heterogeneous feature spaces that encode distinct aspects of the signal [5,6,7].

Several studies have examined feature concatenation and hybrid descriptor construction for EMG classification [5,9]. However, most of these approaches focus on performance improvement without explicitly testing whether the constituent representations provide complementary information [5,7]. As a result, it remains unclear whether observed gains reflect genuine information complementarity or simply increased feature dimensionality [7,27].

This issue is particularly relevant for amputee EMG analysis, where physiological variability may alter both the location and the form of discriminative information within the signal [1,4]. Under such conditions, fixed representations may capture only a subset of the available neuromuscular information [5,6]. Consequently, there is a need for adaptive fusion strategies that integrate complementary signal representations while preserving computational efficiency for real-time prosthetic control [6,8].

To the best of the authors’ knowledge, limited attention has been given to the adaptive fusion of established temporal-spatial EMG descriptors and adaptive decomposition-based representations in amputee motion recognition. Moreover, the extent to which these representation families encode complementary information remains insufficiently understood. This motivates the present study of adaptive multi-representation information fusion for amputee EMG pattern recognition.

## 3. Materials and Methods

### 3.1. Problem Formulation

Let $X\in R^{N\times C}$ denote a multichannel surface electromyography (sEMG) observation, where N represents the number of temporal samples within an analysis window and C denotes the number of recording channels. The objective is to learn a mapping

(1) f:X→Y 

where $Y=\{y_{1},y_{2},\ldots ,y_{K}\}$ represents a finite set of upper-limb movement classes.

This study uses the Ninapro DB3 dataset and the Myo Armband Amputee dataset, which comprise recordings from eleven transradial amputee subjects performing multiple hand, wrist, and grasping gestures [28]. For each analysis window, features are extracted from the acquired EMG signals and then used to infer the corresponding movement intention [4]. The classification task therefore involves identifying the movement class that maximizes the posterior class likelihood given the observed EMG activity [21,29].

Rather than relying on a single feature representation, the proposed framework treats movement recognition as a representation-level information fusion problem. Its aim is to utilize complementary discriminative information encoded in multiple feature spaces to improve class separability and recognition performance while maintaining the computational efficiency required for real-time prosthetic control.

### 3.2. TDPSD Representation

The TDPSD by Khushaba et al. was adopted as one of the feature sets due to its strong discriminatory capability and computational efficiency in myoelectric pattern recognition [9,12,13]. Unlike conventional time-domain descriptors that primarily quantify signal amplitude characteristics, TDPSD captures higher-order temporal dynamics through spectral moment relationships derived directly within the time domain [9,12].

For an EMG segment x(n), three temporal moments are first computed:

(2) $$m_{0}=\sqrt{\sum_{j=1}^{L}x_{j}^{2}}$$

(3) $$m_{2}=\sqrt{\sum_{j=1}^{L}(\Delta x_{j}{)}^{2}}\;$$

(4) $$m_{4}=\sqrt{\sum_{j=1}^{L}(\Delta^{2}x_{j}{)}^{2}\;}\;$$

where Δ and $\Delta^{2}$ denote the first- and second-order discrete difference operators, respectively, $m_{0}$ is the first computed moment, $m_{2}$ is the second computed moment and $m_{4}$ is the third computed moment. *L* is the length of the sample segment and j is the ith sample within the sample segment.

These moments are subsequently combined to derive the six TDPSDs:

(5) $$\log\left(m_{0}\right)\;$$

(6) $$\log\left(m_{2}\right)$$

(7) $$\log\left(m_{4}\right)\;$$

(8) $$\log\left(\frac{m_{0}}{\sqrt{m_{0}-m_{2}}\sqrt{m_{0}-m_{4}}}\right)$$

(9) $$\log\left(\frac{m_{2}}{\sqrt{m_{0}m_{4}}}\right)\;$$

(10) $$\log\left(\frac{m_{4}}{m_{2}^{2}}\right)$$

The resulting feature set provides a compact representation of signal power distribution, frequency-related behaviour, and waveform complexity. Within the proposed framework, TDPSD serves as a stable global descriptor of neuromuscular activity against which the complementary information extracted by the Adaptive LSDL representation can be combined and evaluated.

### 3.3. Adaptive LSDL Representation

The proposed Adaptive LSDL extends the conventional LSDL framework by introducing dynamic localisation-region selection and multi-transform signal decomposition. Unlike the original LSDL methodology, where decomposition is performed using a predefined threshold region, the proposed approach adaptively identifies the most informative localisation region for each signal transformation. This enables the representation to adjust to variations in EMG morphology arising from subject-specific activation patterns, movement-dependent recruitment strategies, electrode placement variability, and signal non-stationarity.

For an EMG segment x(n), four complementary signal representations are first generated:

(11) $$T_{A}=|x\left(n\right)|$$

(12) $$T_{G}=|\nabla x\left(n\right)|$$

(13) $$T_{C}=|\nabla^{2}x\left(n\right)|$$

(14) $$T_{E}=x^{2}\left(n\right)\;$$

Corresponding to the amplitude, gradient, curvature, and energy domains, respectively. Each transform emphasizes a distinct aspect of neuromuscular activity. The amplitude representation captures activation magnitude, the gradient representation highlights local temporal transitions, the curvature representation emphasizes higher-order morphological changes, and the energy representation indicates contraction intensity.

For each transformed signal, Adaptive LSDL performs recursive support localization through a sequence of upper and lower threshold regions centered on a reference level.

Let

(15) $$S_{U}^{\left(k\right)}=\left\{T\left(n\right):T\left(n\right)\geq \tau_{U}^{\left(k\right)}\right\}$$

and

(16) $$S_{L}^{\left(k\right)}=\left\{T\left(n\right):T\left(n\right)\leq \tau_{L}^{\left(k\right)}\right\}$$

denote the upper and lower support regions at localisation depth k, where $\tau_{U}^{\left(k\right)}$ and $\tau_{L}^{\left(k\right)}$ define the corresponding threshold trajectories. As the localization depth increases, the upper thresholds progressively move away from the reference level, while the lower thresholds contract toward lower-valued signal regions, thereby generating a hierarchy of nested support partitions. The localisation thresholds were generated using a fixed threshold increment defined as a proportion of the maximum absolute signal amplitude. Specifically, if M=max∣x∣, the threshold step was defined as Δτ=αM, where α denotes the threshold increment parameter. Successive localisation depths were generated recursively as $\tau_{k+1}^{U}=\tau_{k}^{U}+\Delta \tau ,$ for the upper branch, and $\tau_{k+1}^{L}=\tau_{k}^{L}-\Delta \tau ,$ for the lower branch, producing progressively more restrictive nested support regions until the predefined localisation limit was reached.

Feature vectors are then extracted from each admissible localization region, producing a set of candidate representations.

(17) $$F=\{F_{1},F_{2},\ldots ,F_{K}\}$$

Rather than utilising all localisation regions, an adaptive selection stage is performed independently for each transform, using an embedded classification model. For each signal transform, every admissible localisation region was evaluated independently. Features extracted from each candidate region were classified using the embedded Linear Discriminant Analysis (LDA) model under five-fold cross-validation. The mean validation accuracy associated with localisation depth k was recorded as J(k), and the optimal localisation region was selected according to

(18) $$k^{*}=\arg\underset{k\in K}{\max J\left(k\right)}$$

where K denotes the set of admissible localisation depths. This optimisation was performed independently for the amplitude, gradient, curvature, and energy representations, allowing each transform to select its own optimal localisation region before feature fusion.

This process determines the decomposition region that provides the greatest discriminatory capability for the corresponding transform while avoiding excessive localization, which may remove informative signal structure. Not every localisation depth is considered admissible. Candidate localisation regions are retained only if they satisfy predefined support criteria, including (i) the presence of at least one non-zero support sample within the retained region, (ii) sufficient signal support to permit reliable feature extraction, and (iii) valid computation of all extracted features. Localisation regions failing to satisfy these conditions are discarded and excluded from the optimisation process. The remaining localisation depths constitute the admissible set K. As the number of admissible localisation regions is small, optimisation is performed using exhaustive enumeration rather than iterative or stochastic search, ensuring deterministic and reproducible localisation selection.

**Scheme 1:**
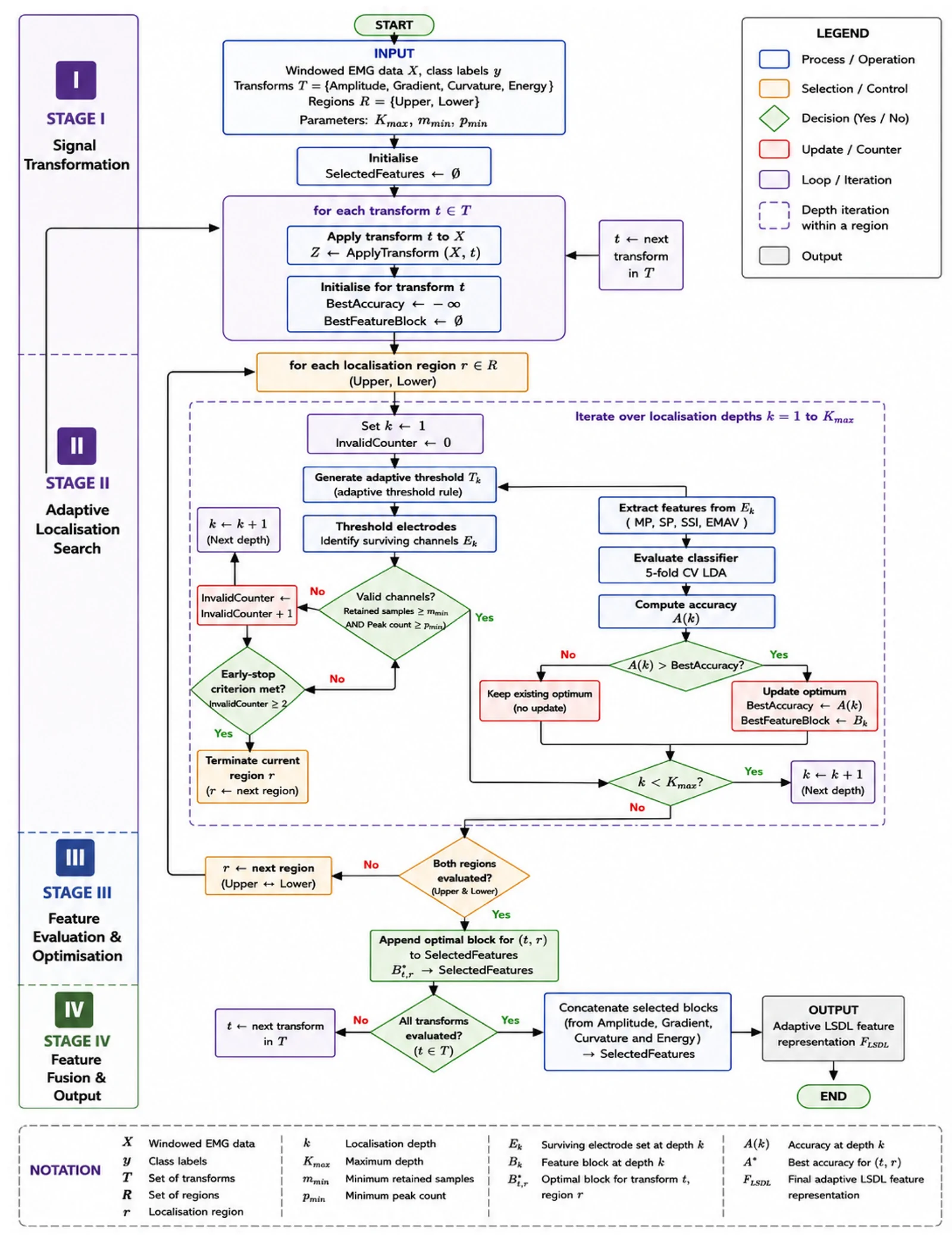
Adaptive LSDL Feature Extraction and Optimisation. A sample Figure of the Adaptive LSDL Localisation Response Curve, can be seen in Figure 2.

A flowchart of the methodology can be seen as follows in Scheme 1:

Let

$$F_{A}^{*},F_{G}^{*},F_{C}^{*},F_{E}^{*}$$

represent the selected feature sets obtained from the optimal amplitude, gradient, curvature, and energy localisation regions, respectively. The final Adaptive LSDL representation is then formed as

(19) $$F_{ALSDL}=[F_{A}^{*},F_{G}^{*},F_{C}^{*},F_{E}^{*}]$$

Consequently, the resulting representation integrates complementary structural information extracted from multiple signal domains while retaining only the most informative decomposition region for each transform. This adaptive selection process reduces redundant features, improves representation efficiency, and provides a compact multidomain feature space that serves as the second information source in the proposed fusion framework.

Following previous work, four signal-based features were extracted at each valid decomposition level: MP (Mean Peak), SP (Sum of Peaks), SSI (Sum of Squared Intensities), and EMAV (Enhanced Mean Absolute Value).

Thus, for a single transform, channel, and localisation level:

(20) $$F_{k}=[\mathrm{MP},\mathrm{SP},\mathrm{SSI},\mathrm{EMAV}]$$

### 3.4. Representation Complementarity

The effectiveness of information fusion depends on the extent to which individual representations capture distinct yet complementary characteristics of the underlying signal. Accordingly, the objective of the proposed framework is not simply to increase feature dimensionality, but to combine representations that encode different sources of discriminatory information.

TDPSD and Adaptive LSDL analyze EMG activity from fundamentally different perspectives. TDPSD characterizes signal behaviour by exploiting higher-order moment interactions; it provides a compact description of the local time-frequency structure of the EMG signal and has demonstrated strong discriminative performance in myoelectric pattern recognition.

In contrast, Adaptive LSDL operates through recursive support localization and adaptive decomposition. Rather than directly describing signal power characteristics, it examines how informative signal structures evolve across multiple localization regions and transformed domains. The amplitude, gradient, curvature, and energy representations reveal distinct morphological characteristics that may not be explicitly captured within the TDPSD feature space. Consequently, Adaptive LSDL captures structural and decomposition-based information associated with signal organization and support geometry.

**Figure 3:**
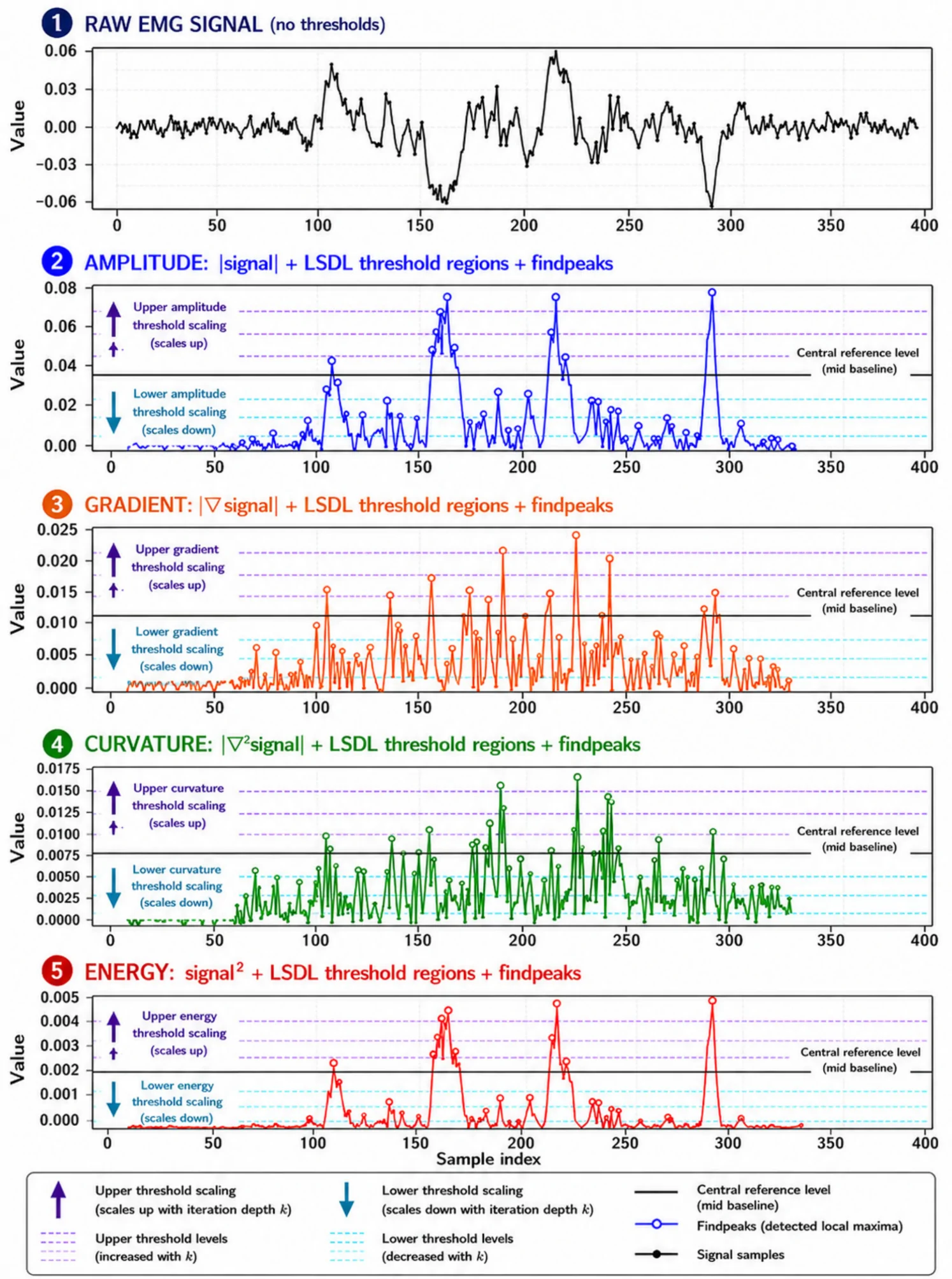
Conceptual Illustration of the Adaptive LSDL.

**Figure 4:**
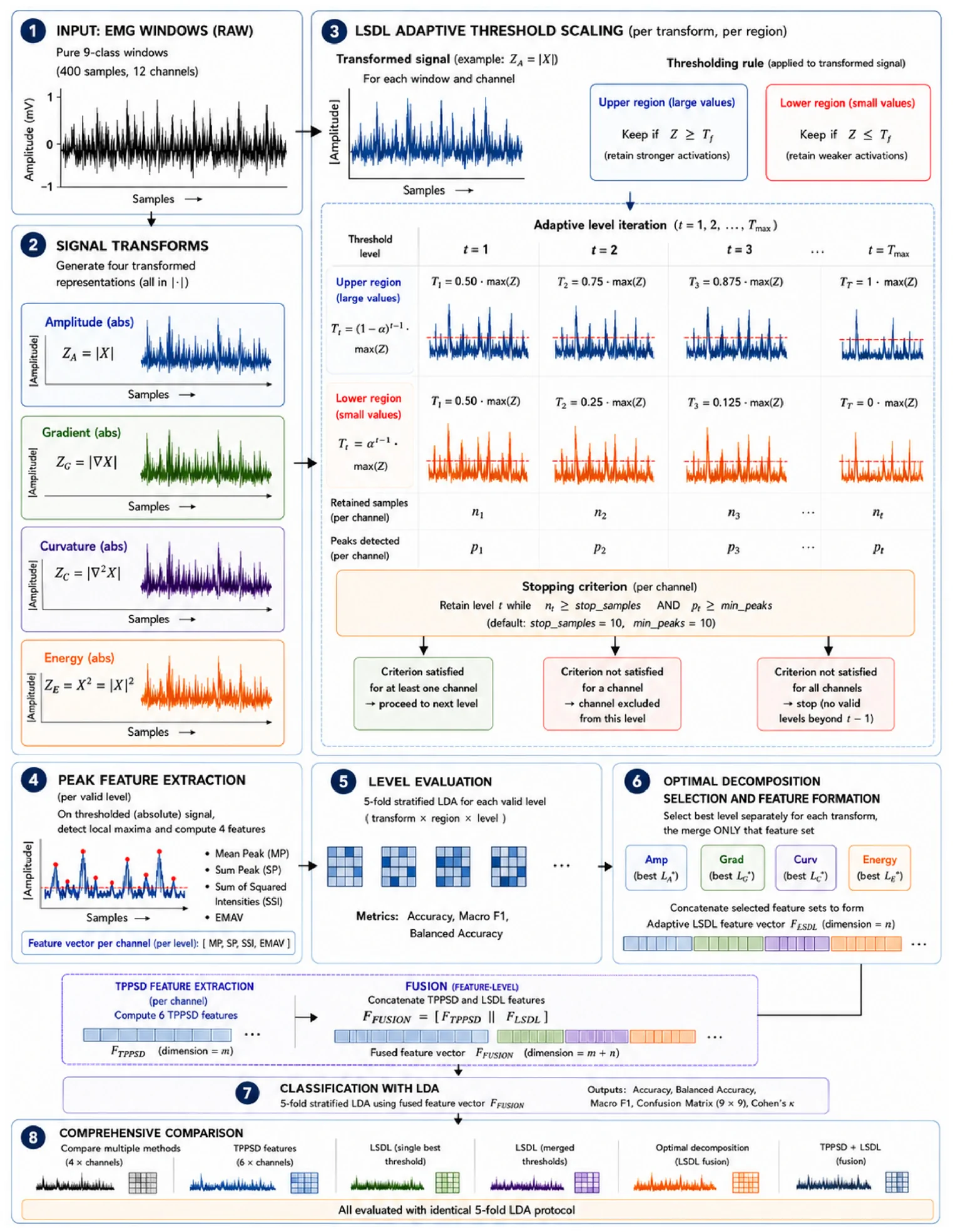
Full proposed model flow. The proposed Adaptive Multi-Representation Fusion Framework classifies movements by integrating TDPSD and Adaptive LSDL feature representations. The complete processing pipeline is shown in Figure 3. After signal acquisition and segmentation, each EMG window is processed simultaneously by two independent representation branches. The first branch extracts the TDPSD feature set, providing a concise description of temporal and spectral signal complexity. The second branch applies the Adaptive LSDL procedure, generating amplitude-, gradient-, curvature-, and energy-based decompositions. From these decompositions, the optimal localization region for each transform is selected to form the final Adaptive LSDL representation.

From an information-theoretic perspective, the two representations may be regarded as complementary feature mappings for $\phi_{T}$ feature mapping for the TDPSD and $\phi_{A}$ feature mapping for the Adaptive LSDL

(21) $$\phi_{T}:X\to F_{TDPSD}$$

and

(22) $$\phi_{A}:X\to F_{ALSDL}$$

where each mapping emphasizes different statistical properties of the same EMG observation. Because the feature extraction mechanisms are derived from distinct signal models, the resulting representations are expected to share only partial information. This limited redundancy increases the likelihood that class-discriminatory information absent from one representation remains available in the other. Feature-level fusion therefore provides a more detailed description of neuromuscular activity than either representation alone. The fused feature space is expected to improve class separability and produce more discriminative decision boundaries. Figure 3 presents a conceptual illustration of the Adaptive LSDL signal thresholding approach, while Figure 4 shows the complementary relationship between TDPSD and Adaptive LSDL. In this figure, the two representations are depicted as distinct but intersecting information sources, whose integration yields an enhanced representation for movement classification.

Let

(23) $$F_{TDPSD}=[f_{1},f_{2},\ldots ,f_{m}]$$

denote the TDPSD feature vector and

(24) $$F_{ALSDL}=[g_{1},g_{2},\ldots ,g_{n}]$$

denote the Adaptive LSDL feature vector. Feature-level fusion is then performed through direct concatenation,

(25) $$F_{Fusion}=[F_{TDPSD},F_{ALSDL}]$$

yielding a unified feature space of dimensionality

(26) d=m+n.

This fusion strategy preserves discriminatory information extracted by both representations while avoiding the information loss that may occur during projection-level or decision-level fusion.

The fused feature vector is then classified using Linear Discriminant Analysis (LDA). LDA was selected because of its established effectiveness in myoelectric pattern recognition and its ability to maximize between-class scatter while minimizing within-class scatter. Because the objective of the proposed framework is to improve representation quality through information fusion, LDA provides an appropriate classifier for evaluating the separability of the resulting feature space without adding modelling complexity.

From a computational perspective, the proposed architecture remains lightweight. TDPSD extraction involves only temporal moment calculations, whereas Adaptive LSDL relies on simple signal transformations and threshold-based support localization. Because no iterative optimization, transform-domain reconstruction, or deep network training is required during deployment, the overall computational burden remains low. Consequently, the framework is suitable for real-time prosthetic control applications while also benefiting from the complementary information provided by both representation branches. The computational pipeline implemented in this study was developed in Python 3.11 using NumPy 2.1.3, SciPy 1.14.1, pandas 2.2.3, and scikit-learn 1.5.2; the corresponding source code and supporting data are provided in the Supplementary Materials.

## 4. Experimental Methodology

### 4.1. Dataset

Experimental validation was conducted using two publicly available electromyography datasets from amputee participants. The primary benchmark dataset was Ninapro DB3, which contains recordings from 11 transradial amputee subjects performing nine gestures, as indicated in the E3 subfile. For each amputee, the performed gestures were thumb flexion, index finger flexion, middle finger flexion, scissor-closing, ring finger flexion, thumb-ring pinch, little finger flexion, whole-hand grasp, and ulnar-side grip [28]. These gestures were performed across multiple force contraction levels. Surface EMG signals were acquired using 12 Delsys Trigno wireless electrodes positioned around the residual limb musculature and sampled at 2 kHz, providing a high-resolution measure of neuromuscular activity.

**Figure 5:**
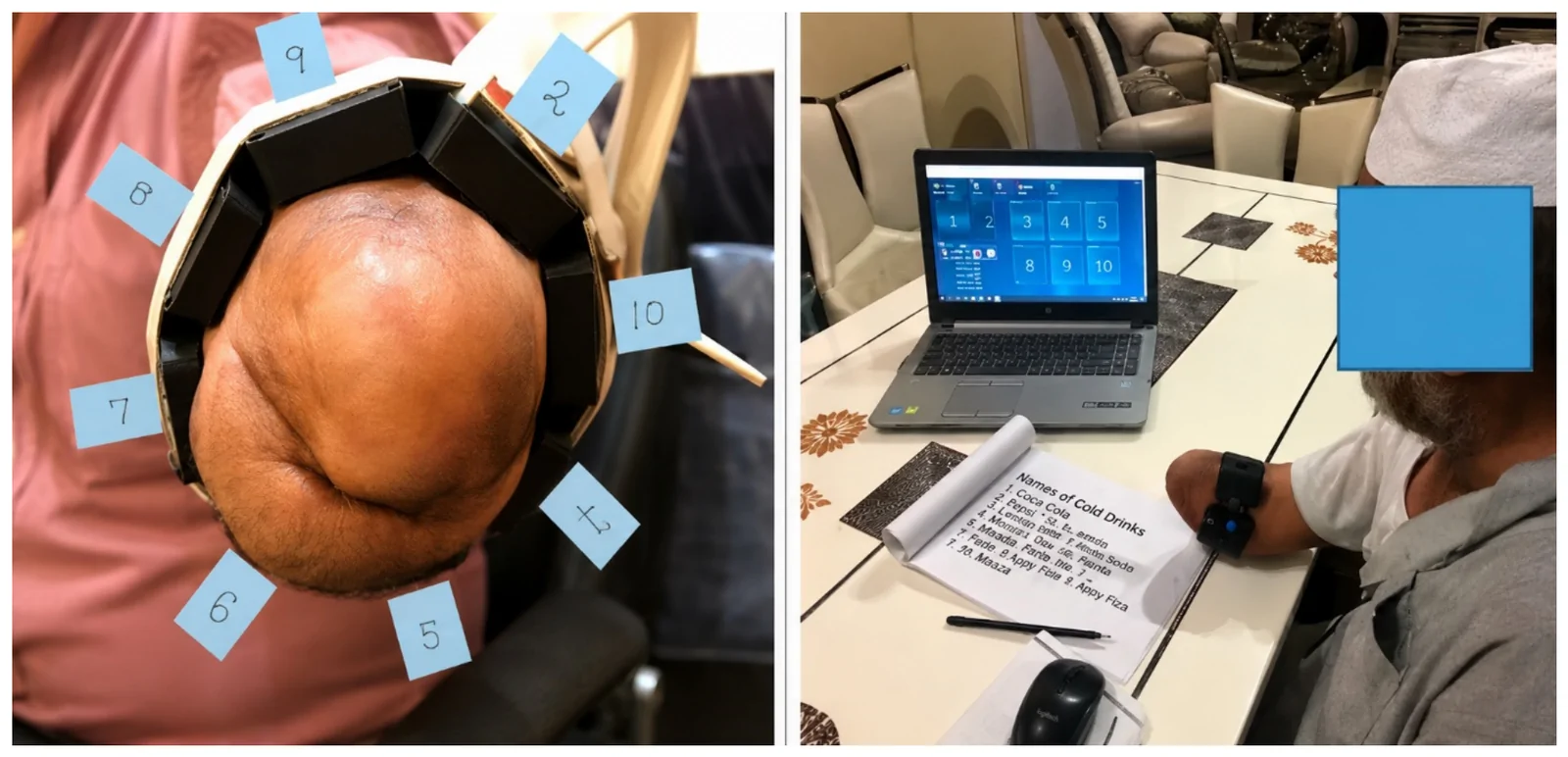
Signal Acquisition Process for the Amputees with the Myo Band, where the numbers in the left figure reflect electrode numbers [30].

To further assess the robustness of the proposed framework under wearable sensing conditions, experiments also incorporated the Transradial Amputee EMG Myo Armband Dataset [30]. This dataset includes recordings from six transradial amputee subjects aged 22 to 60 years who performed 17 predefined hand and wrist gestures: thumb up, extension of index and middle fingers with flexion of the others, flexion of ring and little fingers with extension of the others, thumb opposition to the base of the little finger, abduction of all fingers, fist, pointing index, adduction of extended fingers, wrist supination (middle finger axis), wrist pronation (middle finger axis), wrist supination (little finger axis), wrist pronation (little finger axis), wrist flexion, wrist extension, wrist radial deviation, wrist ulnar deviation, and wrist extension with a closed hand. The signals were acquired using the Thalmic Labs Myo Armband, which consists of eight equally spaced dry surface EMG electrodes positioned circumferentially around the residual forearm and sampled at approximately 200 Hz. An image of an amputee wearing the acquisition device, as well as the acquisition process, is shown in Figure 5.

Collectively, the two datasets provide complementary evaluation scenarios spanning clinical-grade multichannel EMG acquisition and low-cost wearable myoelectric sensing. Their amputee-specific nature introduces substantial inter-subject variability, altered muscle recruitment patterns, limb-loss-dependent activation strategies, and reduced signal consistency, thereby providing a challenging benchmark for evaluating the effectiveness of the proposed adaptive information fusion framework.

### 4.2. Processing Protocol

For the Ninapro DB3 experiments, EMG recordings were segmented into 400-sample windows, corresponding to 200 ms at 2 kHz, with a step size of 400 samples, thus producing non-overlapping windows. Only windows that contained a single gesture label throughout the entire segment were retained for classification. Gesture labels 41 to 49 were remapped to a unified 9-class movement set for evaluation. Feature extraction was applied consistently across all windows using the TDPSD and Adaptive LSDL pipelines, ensuring methodological consistency in the comparative analysis. Candidate Adaptive LSDL decompositions were generated across the amplitude, gradient, curvature, and energy domains, and the optimal localisation region for each transform was selected on the basis of cross-validated classification performance.

For the Myo amputee dataset, signals sampled at 200 Hz were segmented using 40-sample windows (200 ms) with a 20-sample step size (100 ms), resulting in 50% overlap. Each gesture recording was windowed independently, producing labelled samples for the full 17-class classification task. For the MYO dataset, a 50% overlap was adopted because the 200 Hz sampling frequency provides only 40 samples within each 200 ms analysis window. This overlapping segmentation increases the number of available analysis windows while preserving temporal continuity and feature stability, a strategy commonly used in myoelectric pattern-recognition systems.

For the Ninapro DB3 dataset, each repetition yielded approximately 30 analysis windows, resulting in approximately 180 windows per gesture and 1620 windows per participant. For the MYO dataset, each participant performed four repetitions of each of the seventeen gestures. Using 200 ms windows with a 100 ms stride, that is, 50% overlap, each repetition generated approximately 90 to 95 analysis windows, corresponding to approximately 360 to 380 windows per gesture and approximately 6400 windows per participant, depending on recording duration.

As part of the analysis, no additional class-balancing procedures were required, as all classes in both DB3 and MYO were considered sufficiently balanced.

Adaptive LSDL decomposition and TDPSD extraction were performed using the same feature-generation framework used for Ninapro DB3, thereby maintaining consistency across datasets.

A subject-specific evaluation protocol was adopted for both datasets, in which classification models were trained and evaluated independently for each participant. Performance was assessed using 5-fold stratified cross-validation, which preserved class distributions across folds- with approximately 20% of windows allocated to each fold while preserving class proportions.

For the proposed fusion framework, feature vectors from the optimal Adaptive LSDL decomposition and TDPSD representations were concatenated and then classified using Linear Discriminant Analysis (LDA). LDA was selected because of its strong performance in myoelectric pattern recognition, low computational complexity, and suitability for assessing the separability of the resulting fused feature space. The same LDA classifier was used both during Adaptive LSDL localisation optimisation and during the final evaluation of the selected feature representations, ensuring consistency between the optimisation criterion and the subsequent classification stage.

### 4.3. Comparative Methods

A hierarchical benchmarking strategy was adopted to systematically evaluate the contribution of each stage in the proposed framework. The selected methods were designed to assess the incremental benefits of representation engineering, adaptive decomposition, and information fusion.

- Raw Features: represent the lowest complexity baseline and were extracted directly from the segmented EMG windows using statistical descriptors, including mean amplitude, signal sum, sum of squared intensities (SSI), and enhanced mean absolute value (EMAV). This baseline was included to establish the performance achievable without advanced representation learning or signal decomposition.
- TDPSD: employs six time-domain power spectral descriptors per channel, derived from temporal spectral moments. This method was included as a strong conventional EMG representation and serves as the principal benchmark against which the proposed fusion framework is evaluated.
- Original LSDL applies recursive signal decomposition using fixed localisation regions and threshold progression. This benchmark evaluates the effectiveness of decomposition-based feature extraction without adaptive threshold selection and provides a direct comparison with the proposed extension. Yielding 4 features per channel.
- Adaptive LSDL extends the original LSDL by dynamically selecting the optimal decomposition region independently for the amplitude, gradient, curvature, and energy transforms. This comparison isolates the contribution of adaptive localisation and determines whether adaptive decomposition improves discriminatory capability relative to conventional LSDL. Yielding 4 features across 4 transforms per channel i.e 16 features per channel.
- TDPSD + Adaptive LSDL Fusion constitutes the proposed method. Feature vectors obtained from TDPSD and the optimally selected Adaptive LSDL decompositions are concatenated to form a unified representation. This benchmark evaluates whether combining temporal spectral complexity information with adaptive structural decomposition information yields improved class discrimination. Yielding 6 features from the TDPSD and 16 features from the Adaptive LSDL, to produce a concatenation of 22 features per channel.
- Real-Time Convolutional Neural Network (RT-TCNN) was included as a state-of-the-art deep learning benchmark. The RT-CNN was intentionally selected to represent a lightweight real-time deep-learning solution rather than a computationally intensive architecture. In practical prosthetic control systems, inference latency, computational efficiency and deployment on resource-constrained embedded hardware are often as important as absolute classification accuracy. Consequently, the comparison was designed to evaluate whether the proposed handcrafted fusion representation could achieve competitive performance relative to a deployable real-time deep-learning approach. The RT-TCNN utilizes temporal convolutional feature learning directly from EMG signals and provides a contemporary reference against which the proposed handcrafted fusion framework can be assessed [3,7]. Comparing RT-TCNN with the proposed method determines whether the proposed representation-level fusion strategy can achieve competitive performance while maintaining substantially lower computational complexity and improved interpretability [3,22]. This model is a lightweight convolutional neural network that operates directly on rectified EMG windows, receiving 400-sample non-overlapping windows from the 12-channel EMG recordings, with gesture labels 41 to 49 remapped to a unified 9-class classification problem. The architecture consisted of three sequential convolutional blocks comprising Conv1D(12→32, kernel size = 9), Conv1D(32→64, kernel size = 7), and Conv1D(64→64, kernel size = 5) layers, each followed by batch normalization and ReLU activation. Average pooling was applied after the first two convolutional blocks, whereas the final block utilized adaptive global average pooling, followed by a dropout layer (*p* = 0.20) and a fully connected output layer for classification. The resulting architecture contained approximately 39,000 trainable parameters, providing a computationally efficient deep learning baseline. Model training employed the AdamW optimizer with a learning rate of 1 × 10^−3^, weight decay of 1 × 10^−3^, a batch size of 128, and cross-entropy loss for 20 training epochs. Performance was evaluated using stratified five-fold cross-validation, consistent with the experimental protocol adopted for the handcrafted feature-based methods.

Classification performance was primarily assessed using classification accuracy, which provides an overall measure of classification correctness. All models were evaluated using K-fold cross-validation, with K = 5.

## 5. Results

### 5.1. Does Adaptive LSDL Improve Individual Representation Performance?

**Figure 6:**
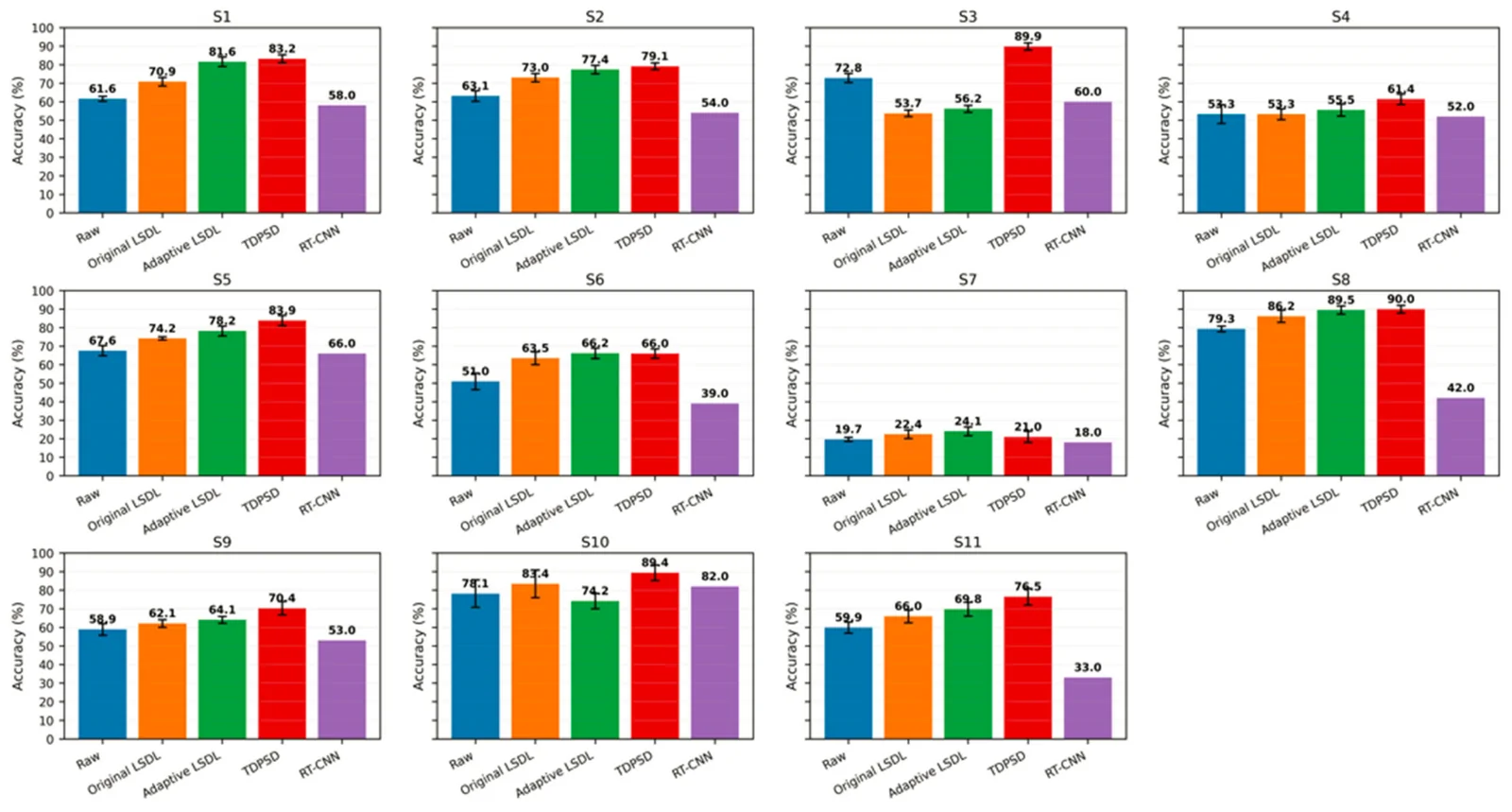
Subject-wise classification accuracies for Raw, Original LSDL, Adaptive LSDL and TDPSD across the DB3 amputee cohort.

**Figure 7:**
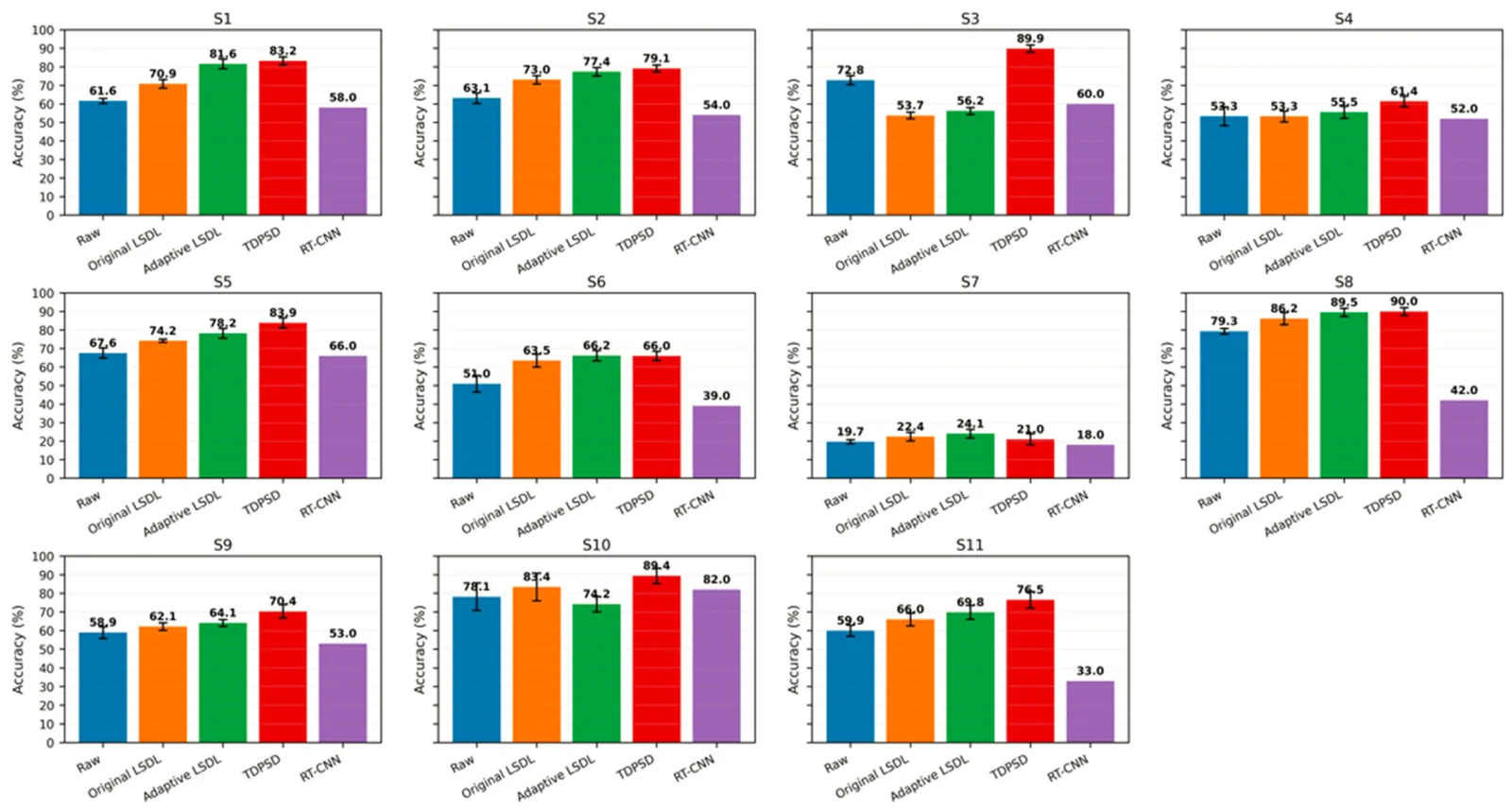
Subject-wise classification accuracies for Raw, TDPSD, Original LSDL, Adaptive LSDL and CNN across the MYO amputee cohort.

The first objective of this study was to determine whether adaptive threshold optimization improves the discriminative performance of the LSDL representation. Figure 6 and Figure 7 show that Adaptive LSDL consistently outperformed both the Raw representation and the original fixed-threshold LSDL representation for most amputees in both datasets. This finding indicates that optimal decomposition characteristics are subject dependent, and that a fixed-threshold structure may fail to capture the most informative signal components.

The observed improvements suggest that adaptive threshold selection enables the decomposition process to align more closely with the underlying neuromuscular activation patterns present in individual EMG recordings. In contrast to the original LSDL framework, which applies a predefined decomposition strategy across all subjects, Adaptive LSDL identifies the transform and threshold region that maximize separability for each amputee. Consequently, the representation is more responsive to subject-specific signal characteristics, including differences in residual musculature, electrode placement, recruitment behavior, and contraction strategies.

Despite these improvements, TDPSD remained the strongest individual representation for many subjects. This result is not unexpected, given that TDPSD was specifically designed to characterize temporal complexity and local spectral morphology in EMG signals.

### 5.2. Does Fusion Improve Recognition Performance?

The principal objective of the proposed framework is not to replace TDPSD, but to determine whether Adaptive LSDL provides complementary information capable of improving movement discrimination when combined with a strong conventional descriptor. Accordingly, the most relevant evaluation is the subject-level fusion gain relative to TDPSD.

**Figure 8:**
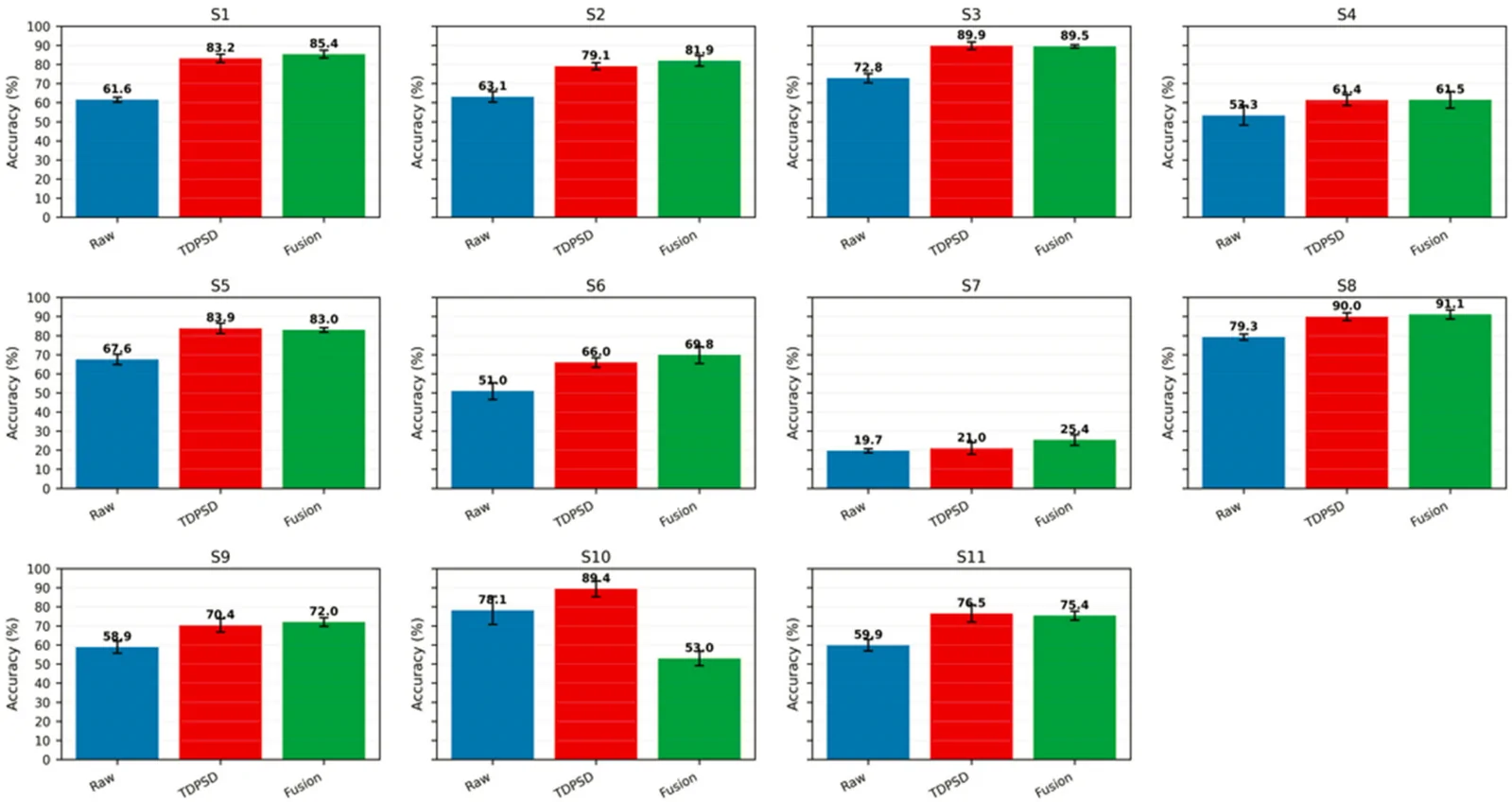
Subject-wise comparison of Raw, TDPSD and TDPSD–Adaptive LSDL Fusion across the DB3 amputee cohort.

Figure 8 shows that, for the DB3 cohort, fusion improved classification performance in 7 of the 11 amputees (63.6%), with gains ranging from 0.13% to 4.39% and a mean improvement of 2.31 percentage points (pp) among subjects who benefited from fusion. The largest improvement was observed for S7 (+4.39%), indicating that the adaptive decomposition features captured discriminatory information that was not represented in the TDPSD representation. Although performance decreased for four subjects, three of these reductions were relatively small (<1.1%). The largest reduction occurred for S10, a special two-gesture task in which adaptive threshold optimization retained only a single electrode segment after feature selection, thereby substantially limiting the available decomposition information. Subject S10 represented a distinct experimental scenario from the remaining participants because, under the Adaptive LSDL optimization, it selected a highly localised representation involving a single electrode channel. This outcome reflected the reduced complexity of the underlying discrimination problem rather than the richer multi-channel activation patterns observed for the other amputees. Consequently, feature-level fusion did not provide the same complementary information available in higher-dimensional classification tasks and instead resulted in degraded performance. For this reason, S10 was excluded from the inferential statistical analysis to preserve comparability across participants who performed substantially different classification tasks. Nevertheless, this observation highlights an important limitation of the proposed framework: the benefits of representation fusion appear greatest when neuromuscular discrimination requires multiple complementary spatial and temporal representations. Future work should investigate Adaptive LSDL under simplified control paradigms to determine whether alternative localis(z)ation or fusion strategies are preferable for low-class-count myoelectric interfaces.

**Figure 9:**
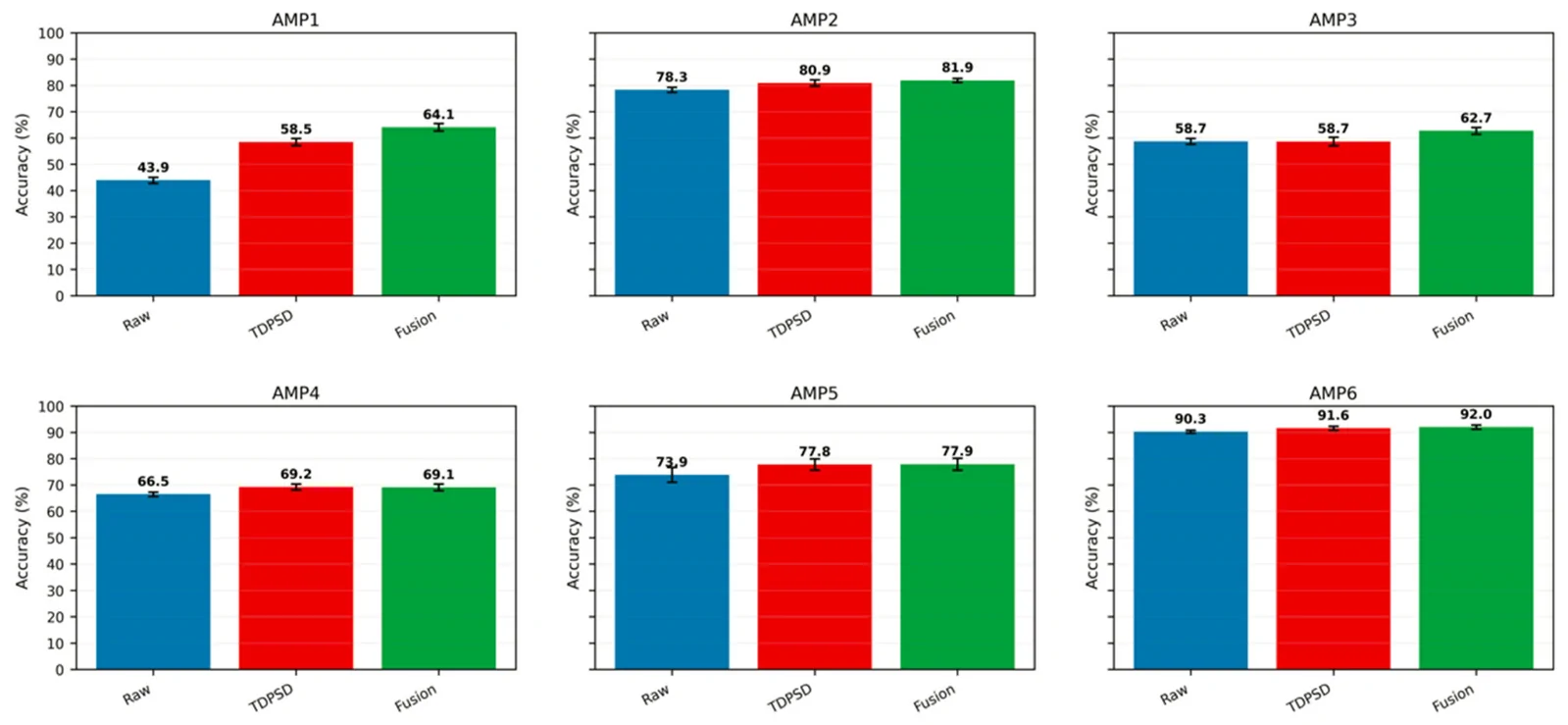
Subject-wise comparison of Raw, TDPSD and TDPSD–Adaptive LSDL Fusion across the MYO amputee cohort.

The MYO cohort showed even stronger evidence of complementarity, as shown in Figure 9. Fusion achieved higher mean classification accuracy in five of the six MYO amputees. However, the magnitude of improvement varied considerably across participants, ranging from negligible changes (e.g., AMP5: +0.12 pp; AMP6: +0.43 pp) to more substantial gains in others. Consequently, the observed positive differences should be interpreted descriptively rather than as evidence of practically meaningful improvement for every individual participant.

Cohen’s *d*z was first calculated across all 11 DB3 amputees, including participant S10, yielding a value of −0.18. This small negative effect reflects the substantial performance reduction observed for S10, which strongly influenced the cohort-level paired differences despite improvements being observed in the majority of participants. To assess the influence of this single participant, a sensitivity analysis excluding S10 was additionally performed, resulting in a Cohen’s *d*z of 0.72, indicating a moderate-to-large effect size. These findings suggest that the apparent benefit of the proposed fusion representation is participant dependent and that cohort-level conclusions are sensitive to substantial individual performance variations.

Likewise, across the six MYO amputees, TDPSD- Adaptive LSDL fusion produced a moderate-to-large improvement over TDPSD alone, with Cohen’s dz = 0.77, supporting the presence of complementary discriminatory information in the adaptive decomposition representation and its ability to improve movement classification performance.

Participant-level paired *t*-tests were performed as a complementary parametric analysis alongside the primary Wilcoxon signed-rank test. Across the complete DB3 amputee cohort, including participant S10, no statistically significant difference was observed between TDPSD and the proposed fusion representation (*t*(10) = −0.589, *p* = 0.569). Given the marked performance reduction for S10, a sensitivity analysis excluding this participant was undertaken to evaluate the influence of this single observation on the cohort-level inference. Under this sensitivity analysis, the fusion representation demonstrated a statistically significant improvement over TDPSD (*t*(9) = 2.277, *p* = 0.049), indicating that the estimated performance gain is sensitive to substantial inter-participant variability. For the MYO amputee cohort, the fusion representation produced a higher mean classification accuracy than TDPSD, although the improvement was not statistically significant (*t*(5) = 1.886, *p* = 0.118). Collectively, these findings support the interpretation that the proposed fusion strategy provides complementary discriminative information, but that its practical benefit is participant dependent rather than uniformly expressed across all amputees.

Importantly, these results should not be interpreted only in terms of average accuracy. The more informative observation is the consistency with which fusion improved performance across subjects. If Adaptive LSDL merely reproduced information already captured by TDPSD, fusion performance would be expected to remain largely unchanged. Instead, improvements were observed in the majority of amputees in both datasets, providing empirical evidence that the two representations encode different aspects of the EMG signal. The fusion results therefore support the complementarity hypothesis presented at the start of the paper and demonstrate that adaptive decomposition features contribute non-redundant discriminatory information beyond that contained in TDPSD alone.

**Figure 10:**
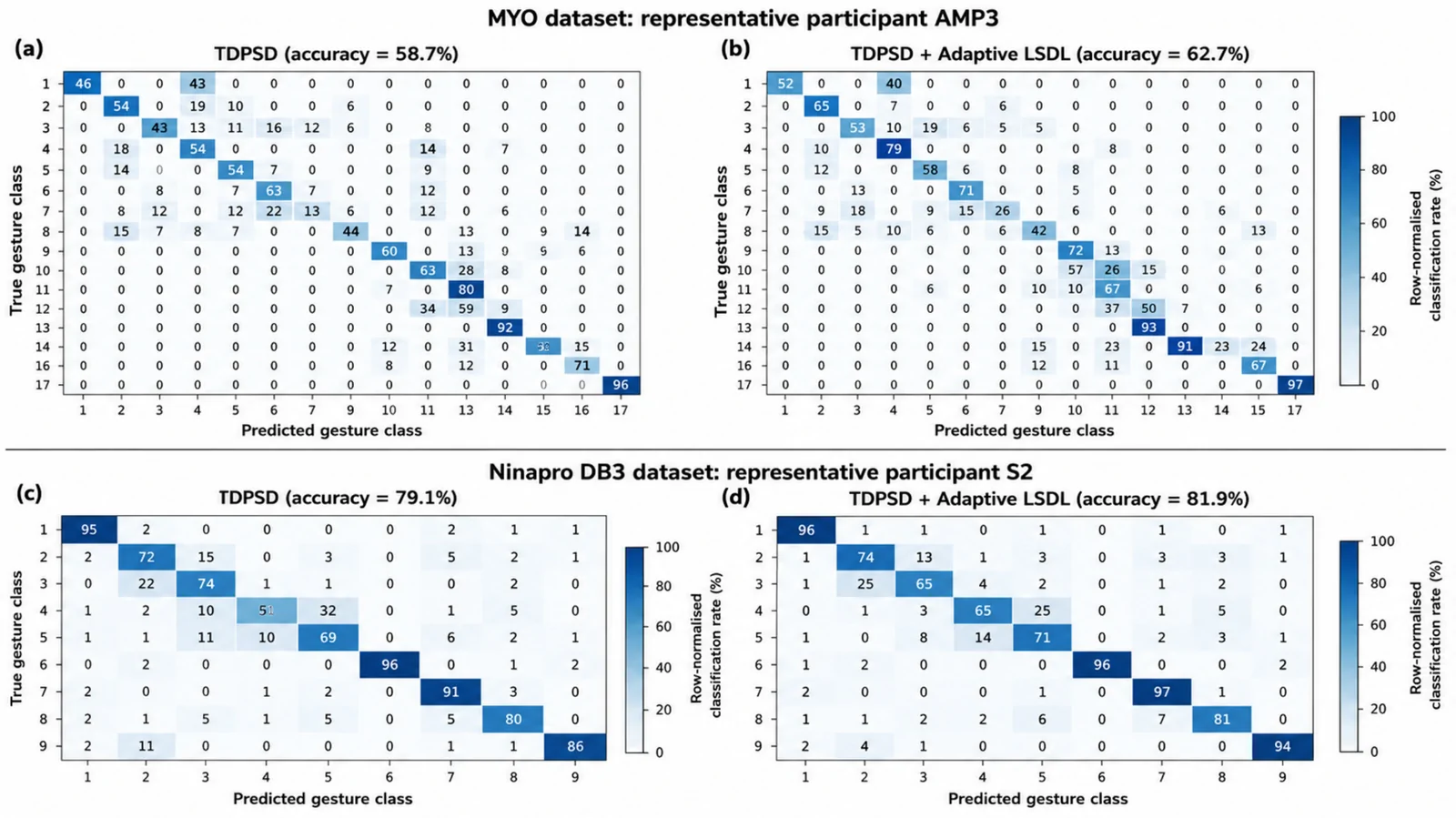
Representative confusion matrices comparing TDPSD with TDPSD + Adaptive LSDL fusion. Row-normalised confusion matrices are shown for representative participants from the Ninapro DB3 dataset (S2; panels (**a**,**b**)) and MYO amputee dataset (AMP3; panels (**c**,**d**)). Each row represents the distribution of predictions for a true gesture class. The fusion framework increased overall accuracy from 79.1% to 81.9% for DB3 S2 and from 58.7% to 62.7% for MYO AMP3, while altering the distribution of errors among specific gesture classes.

To provide further insight into classifier behaviour beyond aggregate performance metrics, representative row-normalised confusion matrices are presented in Figure 10 for one participant from each dataset. The selected participants are representative of the overall cohort and are used solely to illustrate the qualitative classification behaviour of the competing methods. In both datasets, the proposed TDPSD + Adaptive LSDL fusion framework demonstrates increased diagonal dominance relative to TDPSD alone, indicating improved discrimination across multiple gesture classes. Rather than improving a single movement category, the performance gains are distributed across several classes while maintaining high classification performance for movements that we are already well separated. Residual misclassifications occur primarily between biomechanically similar gestures, which is expected given the overlapping muscle activation patterns associated with amputee EMG signals. These representative confusion matrices therefore provide qualitative evidence that the proposed feature fusion improves overall gesture separability and complement the cohort-wide quantitative evaluation and statistical analyses reported in the preceding sections.

#### Accuracy-Derived Information Proxy

Complementary information analysis was conducted on the dataset, focusing on contrasts in the improvements between the TDPSD and Fusion cases, as can be seen in Table 1, Table 2, Table 3 and Table 4, which largely support the qualitative interpretation of the information reported in the preceding sections. Additional tests were performed on the accuracy-derived mutual information lower bound, used as an information transfer proxy, whose formula is as follows;

(27) $$I_{\mathrm{acc}}={log}_{2}(K)+A{log}_{2}(A)+(1-A){log}_{2}\left(\frac{1-A}{K-1}\right)$$

where K = number of gesture classes and A = classification accuracy as a proportion ranging from 0–1.

The results are shown in Table 5. Across both datasets, the fused TDPSD-Adaptive LSDL representation conveyed the greatest estimated class information. For DB3, excluding S10, fusion increased the estimated information from 1.607 bits for TDPSD alone to 1.658 bits. For MYO, it increased from 2.211 bits to 2.301 bits. Although these gains are modest, the consistent increase across two independent amputee cohorts indicates that Adaptive LSDL contributes additional class-discriminative information beyond TDPSD, thereby supporting the hypothesis that the two representations encode complementary neuromuscular structure rather than fully redundant signal information.

Computational times for the candidate methods are reported in Table 6, which presents feature dimensionality, feature-extraction time, classifier inference time, and total processing latency for TDPSD, Adaptive LSDL, and the proposed TDPSD-Adaptive LSDL fusion framework. Despite increasing feature dimensionality to 220 features, the fusion approach incurred only a modest computational cost relative to Adaptive LSDL alone, achieving a total processing time of 2.37 ms per analysis window. The proposed framework exhibits a low computational execution time (2.37 ms per analysis window under the present experimental conditions), indicating that the feature extraction and classification stages are computationally lightweight and compatible with the latency requirements of real-time myoelectric pattern-recognition systems. However, these measurements quantify algorithmic execution time only and do not include system-level delays associated with signal acquisition, buffering, communication or embedded hardware deployment. Consequently, further evaluation on target embedded platforms is required before claims regarding end-to-end real-time prosthetic operation can be made.

Approximate 95% confidence intervals were estimated from the reported five-fold cross-validation means and standard deviations using a Welch variance approximation.

*Only a singular electrode fulfilled the criteria for the Adaptive LSDL for S10, hence the comparatively lower performance.

Values are expressed as percentage points (pp). Approximate 95% confidence intervals were estimated from the reported five-fold cross-validation means and standard deviations using a Welch variance approximation.

## 6. Discussion

### 6.1. Why Does Fusion Work?

The fusion results suggest that TDPSD and Adaptive LSDL characterize fundamentally different properties of the EMG signal. TDPSD was designed to capture local temporal-spectral complexity through descriptors that quantify signal morphology, variability, and dynamic behavior within short analysis windows. Consequently, TDPSD is highly effective for describing local signal structure and remains a strong standalone representation.

Adaptive LSDL, however, operates from a fundamentally different perspective. Rather than characterizing local morphology directly, it decomposes the signal according to adaptive, threshold-defined regions and evaluates transformed signal structures that emerge within those regions. The representation therefore emphasizes how signal energy and activation patterns are distributed across subject-specific decomposition levels. This process captures structural information associated with recruitment organization, activation distribution, and threshold-dependent signal behavior that is largely absent from conventional temporal descriptors.

From an information fusion perspective, the effectiveness of fusion depends not on the individual strength of each representation, but on the extent to which they provide complementary information. Two highly accurate representations may contribute little benefit if they describe the same underlying signal characteristics. Conversely, a weaker representation may substantially improve performance if it captures information absent from a stronger representation. The adaptive threshold analysis supports this interpretation. Different amputees selected different transforms and threshold regions, indicating that the discriminative information captured by Adaptive LSDL varies across subjects and reflects individual neuromuscular signatures rather than a universal decomposition pattern.

The observed fusion gains therefore suggest that TDPSD and Adaptive LSDL occupy different representational spaces. TDPSD primarily characterizes local temporal morphology, whereas Adaptive LSDL characterizes threshold-dependent structural organization. Feature-level fusion combines these complementary perspectives into a unified representation, increasing class separability by providing the classifier with access to multiple independent sources of discriminative information.

Importantly, the fusion improvements were not uniform across all subjects. This variability is expected because the contribution of complementary information depends on individual signal characteristics. For some amputees, TDPSD already captures most of the available discriminative information, limiting the potential gain from fusion. For others, Adaptive LSDL provides access to additional signal structures that substantially improve separability. The fact that fusion improved performance in the majority of amputees across both datasets indicates that these additional structures are frequently informative and supports the broader premise that adaptive decomposition constitutes a valuable supplementary information source for EMG pattern recognition.

Collectively, the results demonstrate that Adaptive LSDL is not merely an alternative feature extraction technique, but a complementary representation capable of exposing subject-specific signal structures that remain inaccessible to conventional temporal-domain descriptors. This ability to reveal and exploit additional information provides the underlying mechanism through which fusion improves recognition performance.

### 6.2. Adaptive Threshold Selection Analysis

**Figure 11:**
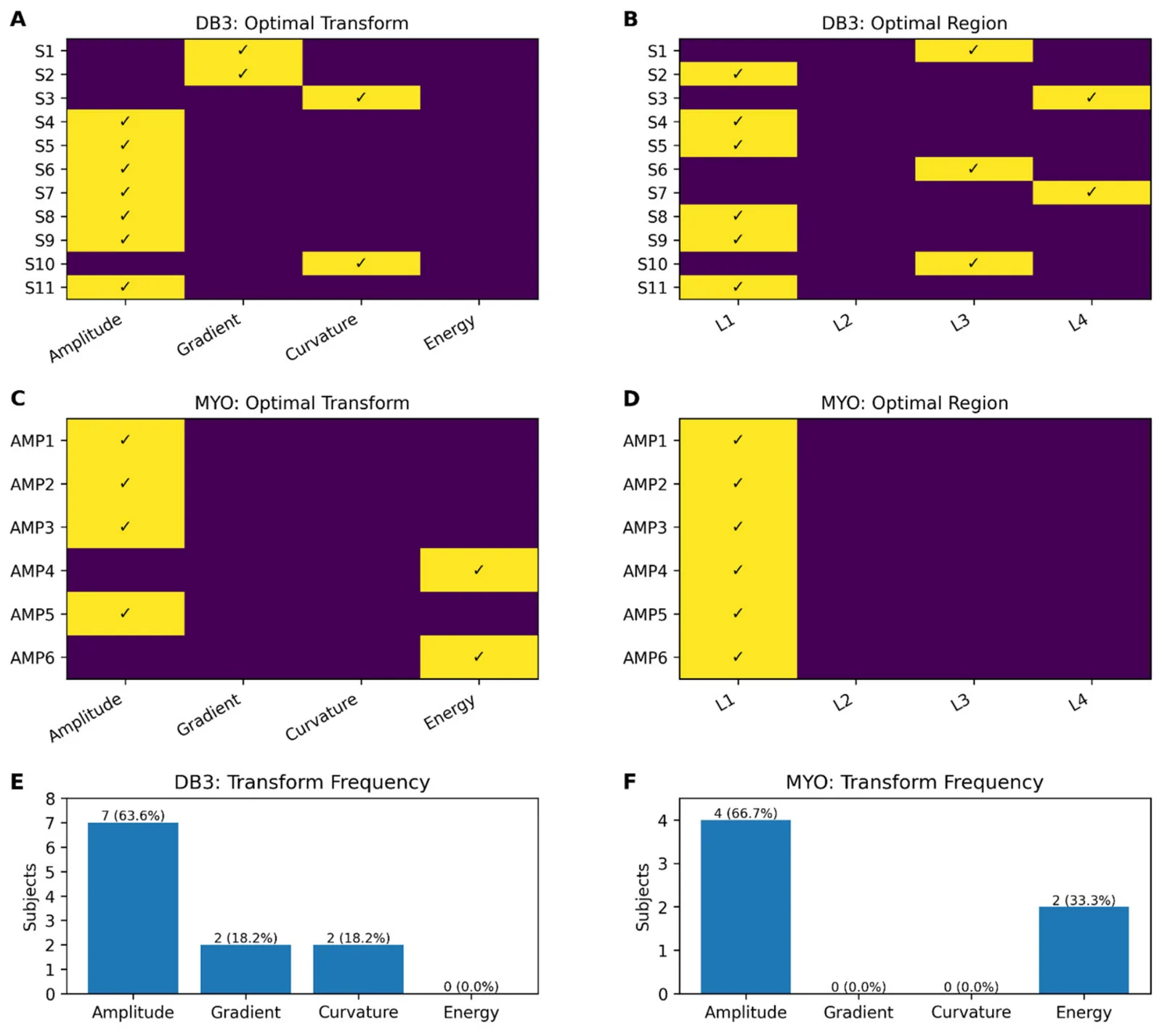
Subject-specific Adaptive LSDL transform and localisation phenotypes across the DB3 and MYO amputee cohorts.

Table 5 and Table 6 summarise the optimal transform–region combinations identified by the proposed Adaptive LSDL framework for the DB3 and MYO amputee cohorts. Several important trends emerge that provide insight into both the discriminative structure of amputee EMG signals and the behaviour of the adaptive decomposition process. The series of plots in Figure 11 show subject specific phenotypes across the DB3 and MYO amputee cohorts. It should be noted that the localisation-response curves were not universally monotonic across participants. Subjects selecting L3 or L4 exhibited local maxima in cross-validated classification accuracy at these deeper localisation depths, whereas others reached their optimum at L1. This behaviour demonstrates that the adaptive optimisation process is identifying subject-specific distributions of discriminative information rather than exhibiting an inherent preference for minimal decomposition. The predominance of L1 reflects the underlying characteristics of many participants’ EMG signals rather than a bias of the optimisation procedure itself.

Panels 11A and 11C present the optimal signal transform selected for each amputee after adaptive decomposition optimisation, Panels 11B and 11D show the corresponding optimal localisation region. Panels 11E and 11F summarise the frequency of transform selection across subjects. Considerable intersubject heterogeneity was seen within the DB3 cohort, with optimal representations distributed across amplitude, gradient, and curvature-based decompositions and across multiple localisation depths (L1 to L4). By contrast, the MYO cohort showed a stronger convergence toward amplitude-based representations and the L1 localisation region. These findings indicate that discriminative EMG information is organised in a subject-specific manner, supporting the premise that Adaptive LSDL identifies personalised neuromuscular signatures and providing a physiological basis for adaptive rather than fixed decomposition strategies.

The most prominent finding was the dominance of amplitude-based decompositions. Within the DB3 cohort, amplitude was selected as the optimal transform for 7 of 11 amputees, whereas the remaining subjects favoured either gradient or curvature representations. Similarly, within the MYO cohort, amplitude was selected for 4 of 6 amputees, with only two subjects favouring energy-based decompositions. Overall, 11 of 17 amputees (64.7%) selected amplitude as the optimal transform, indicating that movement-discriminative information is encoded primarily in the magnitude distribution of the EMG signal rather than in higher-order derivative characteristics. Physiologically, amplitude is closely associated with motor unit recruitment, recruitment synchronisation, contraction intensity, and residual muscle activation levels. The repeated emergence of amplitude-based thresholds therefore suggests that differences in recruitment behaviour remain a primary source of separability in amputee EMG signals.

A second notable finding is the strong preference for lower decomposition regions, particularly L1. Although DB3 showed selections across L1, L3, and L4, all six MYO amputees converged on L1 as the optimal region. Because the adaptive threshold framework partitions the signal according to amplitude-dependent characteristics, selection of lower-level regions implies that informative signal content is concentrated in lower-amplitude activation structures. This suggests that subtle variations in motor unit firing behaviour and background recruitment patterns may provide more discriminative information than large-amplitude muscular bursts. Consequently, classification performance appears to depend more strongly on how muscles are recruited than on the absolute magnitude of contraction.

The results also reveal important differences between datasets. The DB3 cohort showed substantially greater variability, with optimal decompositions spanning amplitude-, gradient-, and curvature-based representations, whereas the MYO cohort showed a much more homogeneous preference for Amplitude-L1, albeit with a much lower sampling frequency. Such differences likely reflect variation in residual limb anatomy, electrode placement, movement complexity, and individual neuromuscular adaptation.

Most importantly, the observed variability shows that no universal optimal decomposition exists across amputees. Different subjects expressed discriminative information through different transforms and threshold regions, supporting the central premise of Adaptive LSDL that EMG signals have subject-specific structural characteristics. These findings therefore provide both physiological evidence of individual neuromuscular signatures and methodological justification for adaptive threshold optimisation, highlighting the limitations of fixed decomposition strategies for amputee EMG pattern recognition.

Although L1 was the most frequently selected localisation depth, particularly within the MYO cohort, the optimisation landscape was not universally monotonic. Participants selecting L3 or L4 exhibited localisation-response curves containing local validation-accuracy maxima at these deeper decomposition levels, demonstrating that discriminative information was concentrated at different threshold regions across subjects. Consequently, the adaptive optimisation mechanism identifies subject-specific optima rather than defaulting to the shallowest admissible decomposition.

### 6.3. Implications for Prosthetic Control

The proposed Adaptive LSDL framework exhibits several characteristics that are desirable for practical myoelectric prosthetic control. Unlike many deep learning approaches that require extensive training data and substantial computational resources, the proposed framework uses lightweight feature extraction and LDA classification, which makes it suitable for embedded and resource-constrained hardware platforms. Because adaptive threshold optimization is performed offline, real-time operation requires only extraction from the selected transform and threshold region, resulting in low computational overhead and minimal latency. Furthermore, the fusion results indicate that additional discriminative information can be obtained without increasing classifier complexity, suggesting a practical pathway to improved prosthetic control performance while maintaining computational efficiency, interpretability, and energy-conscious deployment.

An additional advantage of the proposed framework is its potential to support personalized myoelectric control without retraining computationally intensive deep learning models. The adaptive threshold optimization process enables the signal representation to be tailored to the neuromuscular characteristics of individual amputees, allowing the system to identify the transform and localization region that best captures discriminative information for a given user. This is particularly relevant in amputee populations, where substantial variability exists in residual-limb anatomy, muscle recruitment strategies, electrode placement, and long-term physiological adaptation after limb loss. Rather than learning subject-specific representations through large neural networks that often require extensive calibration data and retraining, Adaptive LSDL provides a lightweight mechanism for adapting the feature space directly. Consequently, the proposed framework may reduce calibration burden while maintaining strong classification performance, offering a practical pathway toward more personalized and clinically deployable prosthetic control systems. A limitation of the present evaluation protocol is that stratified cross-validation was performed at the window level rather than at the recording-repetition level. This choice reflects the intended application of the proposed framework to continuous online prosthetic control, where predictions are generated from temporally adjacent windows extracted from an ongoing EMG stream. Although neighbouring windows are not identical observations and capture evolving neuromuscular dynamics, future work should also investigate repetition-wise and session-wise validation protocols to quantify generalization under increasingly stringent evaluation conditions.

### 6.4. Broader Rehabilitation Implications

Although developed for amputee EMG pattern recognition, the proposed adaptive decomposition framework may have broader relevance in rehabilitation engineering. The underlying principle of subject-specific threshold optimization could be applied to rehabilitation robotics, powered exoskeletons, and assistive control systems that rely on physiological signal interpretation. Similar opportunities may exist for mechanomyography (MMG) and electroencephalography (EEG), in which discriminative information is often distributed across multiple signal components. Emerging applications, such as uterine electromyography (EHG) monitoring for preterm birth assessment, may also benefit from adaptive decomposition strategies. Although these applications remain future directions, the present findings suggest that adaptive threshold selection may offer a general method for extracting subject-specific information from complex biosignals.

### 6.5. Limitations

Several limitations should be acknowledged. First, validation was restricted to the Ninapro DB3 and MYO amputee datasets, and further evaluation in larger, more diverse cohorts is needed to establish generalizability. Second, all experiments were conducted offline; consequently, the effects of Adaptive LSDL on real-time prosthetic control, user adaptation, and closed-loop operation remain unknown. Third, robustness to electrode displacement, socket repositioning, and day-to-day variability was not examined, despite their known effects on EMG-based control systems.

The Adaptive LSDL framework uses identical stratified five-fold cross-validation partitions to evaluate all candidate localization regions, thereby ensuring a fair and consistent comparison among competing localization strategies. The embedded LDA classifier is trained exclusively on the training folds and is evaluated on the corresponding held-out test folds, which helps prevent information leakage during classifier training. Nevertheless, because localization-region selection and performance estimation are both derived from the same cross-validation procedure, a modest optimistic selection bias cannot be entirely excluded. Future work will examine nested cross-validation and independent external validation cohorts to further separate model selection from final performance estimation and to provide an unbiased assessment of generalization performance.

Finally, although fusion improved performance for most subjects, the benefits were not universal, indicating that the contribution of adaptive decomposition is subject dependent. These limitations identify important directions for future work while supporting a balanced interpretation of the reported findings.

## 7. Conclusions

This study introduced Adaptive LSDL, a subject-specific extension of the Local Signal Decomposition Layer framework for EMG-based movement recognition. By adaptively selecting the optimal transform and threshold region for each individual, the proposed approach consistently improved representational quality relative to both the original fixed-threshold LSDL formulation and the raw signal representation. The adaptive threshold analysis further revealed substantial intersubject variability in optimal decomposition characteristics, supporting the need for personalized decomposition strategies in amputee EMG pattern recognition.

The complementary nature of Adaptive LSDL and TDPSD was subsequently examined through feature-level fusion. Across both the DB3 and MYO amputee cohorts, fusion improved performance for most subjects and achieved superior overall recognition performance compared with either representation alone. These findings indicate that Adaptive LSDL captures discriminative structural information that is not fully represented in conventional temporal-domain descriptors. Furthermore, the proposed fusion framework consistently outperformed the RT-CNN benchmark while requiring substantially fewer trainable parameters and less computational resources.

Importantly, these performance gains were achieved using a parsimonious architecture comprising lightweight feature extraction and LDA classification, making the framework suitable for real-time and embedded deployment scenarios. Collectively, the results demonstrate that adaptive threshold decomposition is an effective mechanism for identifying subject-specific EMG information and that its integration within an information fusion framework can enhance movement recognition performance while maintaining computational efficiency suitable for practical prosthetic control applications.

## Figures and Tables

**Figure 2:**
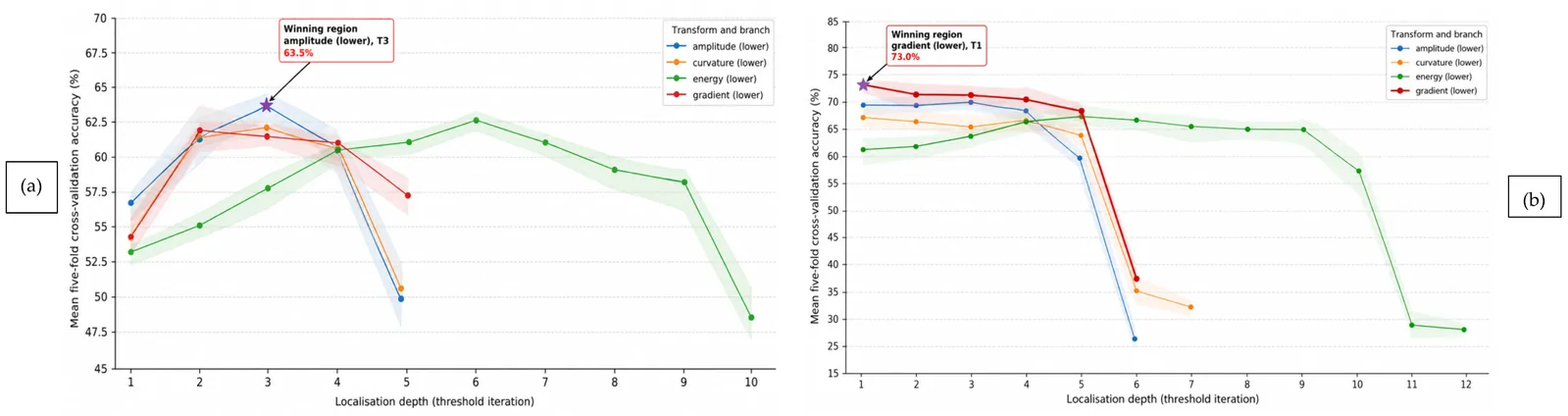
Representative Adaptive LSDL localisation-response curves illustrating subject-specific optimisation behaviour. (**a**) Example participant exhibiting a local maximum at an intermediate localisation depth (T3), demonstrating that deeper localisation can provide the greatest discriminative performance. (**b**) Example participant whose optimal performance occurs at the shallowest localisation depth (T1), illustrating that localisation-response profiles differ across individuals. Together, these examples demonstrate that Adaptive LSDL identifies subject-specific optima rather than exhibiting an inherent preference for minimal decomposition. Following optimisation, only the feature vector extracted from the optimal localisation region of each transform is retained.

**Table 1:** Fusion-Outcome for DB3.

Participant	TDPSD	Fusion	Difference (pp)	Approx. 95% CI (pp)
S1	83.22	85.43	+2.22	−0.26 to +4.69
S2	79.13	81.92	+2.79	−0.02 to +5.59
S3	89.86	89.49	−0.36	−2.29 to +1.56
S4	61.39	61.52	+0.13	−4.33 to +4.59
S5	83.86	83.04	−0.82	−3.52 to +1.88
S6	66.02	69.84	+3.83	−0.57 to +8.23
S7	21.02	25.41	+4.39	+0.95 to +7.84
S8	89.97	91.10	+1.13	−1.49 to +3.75
S9	70.35	72.00	+1.65	−2.00 to +5.30
S10	89.43	52.98	−36.44	−41.07 to −31.81
S11	76.47	75.40	−1.08	−5.45 to +3.30

**Table 2:** Summary statistics of TDPSD–Adaptive LSDL fusion performance improvements across the DB3 amputee cohort.

Metric	Value
Subjects improved	7/11 (63.6%)
Largest gain	+4.395 percentage points (S7)
Mean gain (improved subjects only)	+2.305 percentage points

**Table 3:** Fusion-Outcome for Myo.

Participant	TDPSD (%)	Fusion (%)	Difference (pp)	Approx. 95% CI (pp)
AMP1	58.48	64.07	+5.60	3.99 to 7.21
AMP2	80.94	81.91	+0.97	−0.24 to 2.18
AMP3	58.67	62.71	+4.04	2.30 to 5.78
AMP4	69.25	69.13	−0.12	−1.55 to 1.31
AMP5	77.80	77.93	+0.12	−2.45 to 2.69
AMP6	91.61	92.04	+0.43	−0.48 to 1.34

**Table 4:** Summary statistics of TDPSD–Adaptive LSDL fusion performance improvements across the MYO amputee cohort.

Metric	Value
Subjects improved	5/6 (83.3%)
Largest gain	+5.599 percentage points (AMP1)
Mean gain, improved subjects only	+2.231 percentage points

**Table 5:** Accuracy-Derived Class Information Transfer Across Feature Representations (bits).

Dataset	Adaptive LSDL	TDPSD	Fusion	Fusion—TDPSD
DB3, S10 excluded	1.334 bits	1.607 bits	1.658 bits	+0.050 bits
MYO	1.927 bits	2.211 bits	2.301 bits	+0.090 bits

**Table 6:** Computational complexity and inference latency of the evaluated EMG representations.

Method	Feature Dimensionality	Feature Extraction (ms/Window)	LDA Inference (ms/Window)	Total Latency (ms/Window)
TDPSD	72	0.25	0.0004	0.25
Adaptive LSDL	148	2.15	0.0008	2.15
Fusion	220	2.37	0.0011	2.37

## Data Availability

Data is available from a cited repository within the manuscript.

## Supplementary Materials

The following supporting information can be downloaded at: https://www.mdpi.com/article/10.3390/bioengineering13080900/s1.
